# Hybrid fractional Fourier transform for filtering and imaging in a single digital holography workflow

**DOI:** 10.1364/OL.575799

**Published:** 2025-12-15

**Authors:** Muge Topcu, Serhat Tozburun

**Affiliations:** 1 Izmir Biomedicine and Genome Center, Izmir 35340, Turkey; 2 Izmir International Biomedicine and Genome Institute, Dokuz Eylul University, Izmir 35340, Turkey; 3 Department of Biophysics, Faculty of Medicine, Dokuz Eylul University, Izmir 35340, Turkey

## Abstract

We propose a hybrid fractional Fourier transform (hybrid FrFT) framework for off-axis digital holographic microscopy. This framework combines optical FrFT, implemented via a novel, to the best of our knowledge, three-lens object arm design, with digital FrFT filtering into a unified process. By replacing all FFT operations with FrFT, our approach adaptively minimizes conjugate overlap in spatial frequency distribution and improves the recovery of higher-frequency diffraction components. Both simulations and experimental results reveal significant enhancements in the resolution of amplitude and phase reconstruction while adhering to practical optical design constraints.

Off-axis digital holographic microscopy (DHM) is a quantitative phase imaging technique that enables single-shot, non-invasive, and label-free imaging of transparent samples [1]. It records intricate digital holograms by interfering object and reference waves at an off-axis angle, producing diffraction patterns that contain both zero-order and ±1 order terms. By isolating one diffraction order (either real or imaginary conjugates) in the Fourier domain, the object’s complex wavefront can be re-constructed to obtain phase and amplitude information [2,3]. The reconstruction quality depends largely on effective diffractionorder separation, which is enhanced using methods such as zero-padding, zero-order suppression, background subtraction, and spatial bandwidth optimization [4–7].

A recent approach to improving resolution and reducing noise in DHM employs the fractional Fourier transform (FrFT), which is implemented both optically [8] and digitally [4]. The FrFT extends the traditional Fourier transform by introducing a continuous transform order (), corresponding to a rotational angle (*∅* = *απ*/2) in optical wave propagation [9]. Under the Fresnel approximation, optical field propagation can be modeled as an FrFT of the source field [10]. This added degree of freedom makes the FrFT especially valuable in digital holography, as it enables flexible control of diffraction orders in fractional Fourier domains.

In DHM, the digital implementation of FrFT (digital FrFT) enhances resolution compared to conventional Fourier transform filtering by filtering diffraction orders in the fractional Fourier domain, rather than in the conventional Fourier domain. It also simplifies hologram processing, making it a powerful tool for modern imaging systems and effective in both telecentric and non-telecentric configurations [5,11]. By contrast, optical FrFT records holograms directly in the fractional Fourier plane and can achieve higher resolution; however, its performance is limited by practical optical design constraints [8,12].

tudy introduces a hybrid FrFT framework that integrates optical and digital implementations o ction resolution (Fig. 1). A three-lens design applies the FrFT in the object arm while preserving magnification, and digital FrFT is used during filtering. Extending FFT to the FrFT domain enables greater flexibility in wavefront manipulation and adaptive resolution tuning within physical constraints. The hybrid approach facilitates recovery of high- frequency diffraction components by creating an asymmetric spatial frequency distribution that reduces overlap with DC and conjugate terms. improves both amplitude and phase reconstruction in off-axis DHM.

The recording section, as shown in Fig. 1, utilizes an off-axis DHM setup based on a Mach–Zehnder interferometer. Illumination is provided by a 635 nm diode laser, split 50/50 by a fiber coupler. The object arm includes a collimator for uniform illumination, a microscope objective (*f* = 4.5 mm, *NA* = 0.75)) paired with a tube lens (*f* = 35 mm)), and a movable fractional lens (*f* = 75 mm)) for optical FrFT implementation. The reference arm consists of a beam expander (L1, L2) along with a collimator.

For hologram acquisition, a beam splitter collects the object (O) and 3° tilted reference (R) beams, and the image is formed on a CMOS camera (1280 × 1024 pixels, 5.3 μm pixel size, Thorlabs, USA). A fractional lens positioned between the camera and tube lens enables the implementation of optical FrFT. The optical fractional order (*a_o_*) is determined using the focal length of the fractional lens (*f*) and the distances to the tube lens (*d*_1_) and CMOS sensor (*d*_2_), as outlined in Eq. (1) [8,9]:

(1) $$\phi_{o}=\arccos\left(\frac{\sqrt{f-d_{1}}\sqrt{f-d_{2}}}{f}\right),a_{o}=\phi_{o}\frac{2}{\pi }.$$

The intensity distribution (*I*) in the object arm with an optical FrFT $\left(F^{a_{o}}\right)$ of order *a_o_* can be expressed as follows:

(2) $$I=\left({\left|F^{a_{o}}\{O\}\right|}^{2}+|R{|}^{2}\right)+F^{a_{o}}\{O\}R^{*}+{\left\{F^{a_{o}}\{O\}\right\}}^{*}R.$$

In the filtering section, one of the ±1 diffraction orders (either the third or fourth term) is isolated from the DC component (comprising the first and second terms) along with its conjugates. This process minimizes information loss, as described in Eq. (2). To further optimize this separation, a digital FrFT is applied to the recorded intensity. This technique efficiently rotates the spatial frequency content, resulting in a hybrid FrFT $\left(F^{a_{h}}\right)$ characterized by the order of *a_h_* = *a_d_* ± *a_o_* that correctly integrates the relevant diffraction terms. A region of interest is then carefully selected to isolate the chosen diffraction term. Following this, the filtered diffraction order term is transformed into the frequency domain using the 1 − *a_h_* order FrFT. Thereafter, the frequency domain representation of the filtered diffraction order is centered to eliminate the carrier frequency. Subsequently, this centered frequency domain representation is reverted to the spatial domain through an inverse Fourier transform, resulting in an out-of-focus complex field. The out-of-focus complex field is then centered and propagated to obtain the in-focus complex field. Ultimately, both amplitude and phase images can be retrieved from the in-focus complex field. For further mathematical explanation, see Supplement 1.

Initially, a simulation study was conducted to visualize the mathematical relationship between optical, digital, and hybrid FrFTs. The amplitude distribution of the object wave was based on a USAF-1951 test pattern. Similar to conventional systems, the simulation aimed to replicate the typical process of recording a hologram in the spatial domain (*a_o_* = 0, *a_d_* = 0) and then filtering it in the frequency domain (*a_o_* = 0, *a_d_* = 1). This process is referred to as the digital FrFT, where *a_o_* = 1 and 0 < *a_d_* < 1. Conversely, recording in the FrFT domain and filtering in the frequency domain is referred to as the optical FrFT, where the interval is 0 < *a_o_* < 1 and *a_d_* = 1. The simulation proceeded as follows: The object wave was zero-padded to ensure that it had the minimum spatial bandwidth required for conjugate separation. The object wave was then propagated using FrFT [13]. A plane reference wave was generated with a specific tilt angle. The interference between the object wave and the reference wave resulted in an off-axis hologram. To achieve DC suppression, Laplacian convolution was used. All simulations and numerical processes were performed using MATLAB.

Figure 2 shows the space-frequency domain matrices of simulated off-axis holograms of the USAF-1951 target. The horizontal axis represents the optical FrFT (*a_o_* ranges from 0 to 1), and the vertical axis represents the digital FrFT (*a_d_* ranges from 0 to 1). The first row of the matrix depicts the spatial domain corresponding to the intensity distribution, while the last row represents the frequency domain. Intermediate rows, where the optical and digital fractional orders lie between 0 and 1 (but are not equal), as shown by the purple dashed square, are referred to as hybrid FrFT. As expected, the symmetric growth of the conjugates from a fixed center is calculated as a function of the optical fractional order. Meanwhile, the digital FrFT causes a center shift towards the DC component. In contrast, the hybrid FrFT results show asymmetric growth in both size and position.

Figure 3 compares the reconstruction resolution of a sample amplitude in off-axis holography simulations using optical, digital, and hybrid FrFT models. In panel (a), we present simulated reconstructions created from combinations of five optical fractional orders (*a_o_* = [0, 0.2, 0.5, 0.8, 1]) and four digital fractional orders (*a_d_* = [0.2, 0.5, 0.8, 1]). Each reconstruction is framed and color-coded to indicate its corresponding domain: conventional FT (gray), optical FrFT (blue), digital FrFT (green), and hybrid FrFT (purple). The insets provide a zoomed-in view of USAF Group 6, focusing specifically on the third region of the line pair element. The reconstructed profile is highlighted in red, contrasting with the reference profile shown in a dashed line. Above each image, the corresponding root mean square error (RMSE) emphasizes the accuracy of the reconstructions. Panel (b) includes a detailed table outlining the space-frequency locations of the diffraction orders utilized in each reconstruction from panel (a), organized following the established color codes. The hybrid fractional orders (*a_h_*), derived from the −1 diffraction orders, are depicted on a gradient scale. As *a_h_* approaches 0, the frame color whitens, highlighting the benefits of the hybrid approach. The isolated −1 diffraction orders are placed in the upper-left corners of the matrices for each optical and digital fractional order pair (*a_o_*, *a_d_*) shown in Fig. 2. The value of *a_h_* is directly related to the maximum high-frequency components that can be reconstructed from the diffraction order in the corresponding space-frequency matrix achieved through hybrid FrFT. As *a_h_* shifts from 1 to 0, the diffraction order’s distribution in the space-frequency matrix allows better isolation of high frequency components, resulting in improved reconstruction’s resolution. Panel (c) shows the USAF-1951 test target as a critical reference for evaluating the resolution. This simulation analysis highlights the effectiveness of our approaches and establishes a benchmark for experimental results.

In panel (d), we present normalized values for RMSE and structural similarity index measure (SSIM) to reveal resolution trends. The solid lines represent combinations with a fixed *a_d_* value of 1, while red and green dots indicate various *a_d_* values paired with each *a_o_*. The shaded regions cover all possible pairs (*a_o_*, *a_d_*). As expected, RMSE decreases as *a_o_* approaches 1, signifying an enhancement in resolution. The combination of digital FrFT and optical FrFT enhances resolution up to *a_o_* value of 0.5, after which point RMSE begins to rise. Similarly, higher SSIM reflects increased fidelity but also declines beyond *a_o_* value of 0.5. We combine RMSE and SSIM into a single metric, color-coded from black (lowest) to pink (highest), making it easier to interpret quality scores. This heatmap illustrates those *a_o_* values below 0.5, when paired with increasing digital fractional orders (bottom-up), significantly enhance the combined score. The pairing at *a_o_* = 0.5, *a_d_* = 0.5 marks a turning point: digital FrFT improves resolution up to 0.5 fractional order, while decreasing the resolution beyond this threshold. For higher *a_o_* values (0.8 and 1), any digital FrFT results in a lower combined score. A clear correlation exists between decreasing combined scores and rising *a_h_* value (panel b), indicating improved reconstruction’s resolution as *a_h_* approaches 0. Combinations with higher *a_o_* consistently achieve superior scores, emphasizing the prominent role of optical FrFT. Thus, this heatmap serves as a practical guide for optimizing the combined score of both optical and digital FrFTs, allowing for effective resolution enhancement.

In experimental recordings using the DHM setup shown in Fig. 1, we utilized a Newport USAF High-Res1 test target to perform hologram acquisitions and image reconstructions. We explored several methods, including conventional FT for *a_o_* = 0 *a_d_* = 1, digital FrFT for *a_o_* = 0 *a_d_* = 0.6, optical FrFT for *a_o_* = 0.2 *a_d_* = 1, and ultimately, hybrid FrFT for *a_o_* = 0.2 *a_d_* = 0.8. The optical FrFT was executed by adjusting the fractional lens position within the object arm, as defined in Eq. (1). The space-frequency domains of the holograms are shown in Fig. 4(a). Amplitude reconstructions are presented in Figs. 4(b) and 4(c), along with enlarged views (the background displayed at the bottom). Plot profiles for Group 9, line pair element 2 (0.87 μm bar width) are compared in Fig. 4(d) alongside key non-reference metrics such as standard deviation (STD), coefficient of variation (CV), and mean absolute deviation (MAD) in Fig. 4(e). The results show significant improvements in resolution and noise reduction when switching from conventional FT to hybrid FrFT, successfully resolving the smallest line-pair in the test target. By leveraging a three-lens design, we have transcended the limitations of fractional lenses, achieving submicron resolution in hybrid FrFT mode. This accomplishment exemplifies the efficacy of our approach and its potential for transformative advancements in optical imaging quality.

Phase imaging was performed on adherent HT-29 epithelial cells at 1 day and 4 days post-attachment. One-day-old cells exhibited a distinct morphology, while the four-day-old cells formed characteristic morphological clusters. For accurate reconstructions, we employed the same FrFT combinations optimized with the USAF test target. Figures 5(a)–5(c) show amplitude and phase reconstructions of four-day-old clusters, and panels (d)–(f) show those for the one-day-old cells. Pseudo-3D phase maps in panels (c) and (f) indicate unwrapped phases normalized between 0 and 1. The spatial phase plots for both timepoint groups are in panels (g) and (h). Our qualitative analysis, combined with the plot profiles, indicates that the four-day-old clusters appear more disintegrated under conventional FT. In contrast, they exhibited an increasingly compact and integrated morphology when assessed using digital, optical, and hybrid FrFTs, respectively. The analysis of quantitative background noise shows the following standard deviations: conventional FT = 0.0285, digital FrFT = 0.0255, optical FrFT = 0.0241, and hybrid FrFT = 0.0218, confirming significant noise reduction during phase reconstructions. While digital FrFT provided modest improvements in resolution, optical FrFT showed sharper boundaries in the one-day cells, and even higher resolution was achieved with hybrid FrFT. The transition from conventional FT to hybrid FrFT across all reconstruction modalities greatly enhances the ability to distinguish individual cells, highlighting the superior effectiveness of our approach.

The experimental results in Fig. 4 are consistent with simulations using conventional FT, digital FrFT, optical FrFT, and hybrid FrFT approaches. Both individual and combined FrFT methods improve reconstruction resolution. Small increment optical fractional orders (ranging from 0 to 0.2) achieve notable improvements in resolution and reduce optical design constraints. The proposed three-lens design allows 20X magnification by separating magnification from the FrFT operation, unlike single-lens optical FrFT systems. Digital FrFT provides only marginal resolution enhancement (maximum resolvable line pair group 9, element 1), while optical FrFT offers significantly greater gains (maximum resolvable pair group 9, element 2). The hybrid FrFT approach further enhances resolution and effectively addresses system limitations.

Several limitations should be noted. First, the setup requires identical focal lengths for the tube and fractional lenses, as mismatched lenses can cause scaling effects in holograms. Second, achieving an optical FrFT that enhances resolution is challenged by intensity differences with the reference arm. However, minor fractional-order adjustments still yield notable improvements. Finally, effective digital processing (zero padding and Laplacian convolution) is crucial to overcome physical limits like sensor size, off-axis angles, and numerical aperture.

In conclusion, the hybrid FrFT framework integrates optical and digital FrFT to enhance reconstruction resolution within DHM’s physical constraints. It achieves high magnification with submicron resolution, implemented with a movable lens in the object arm. The work demonstrates, for the first time, improved phase imaging resolution in DHM using optical FrFT. The framework promises a systematic, customizable, and cost-effective pathway for enhanced biological specimen analysis.

## Supplementary Material

**Supplemental document**. See Supplement 1 for supporting content.

Supplemental document

## Figures and Tables

**Fig. 1:**
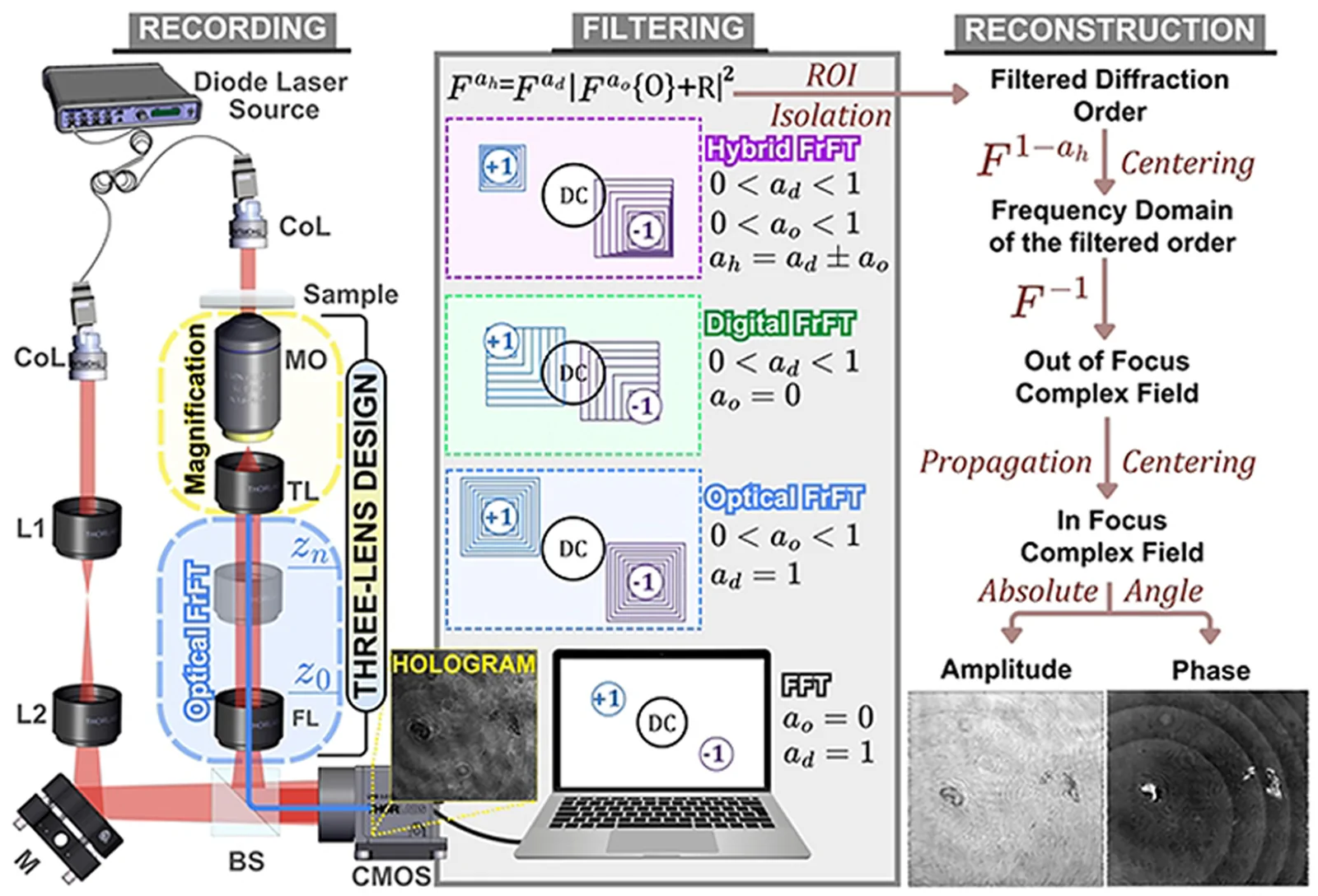
Schematic representation of the Hybrid FrFT workflow for off-axis DHM is described in three stages: a recording section that involves optical FrFT implemented through a three-lens system, a filtering section that applies digital FrFT, and a reconstruction section that includes spectrum shifting, centering, and spreading.

**Fig. 2:**
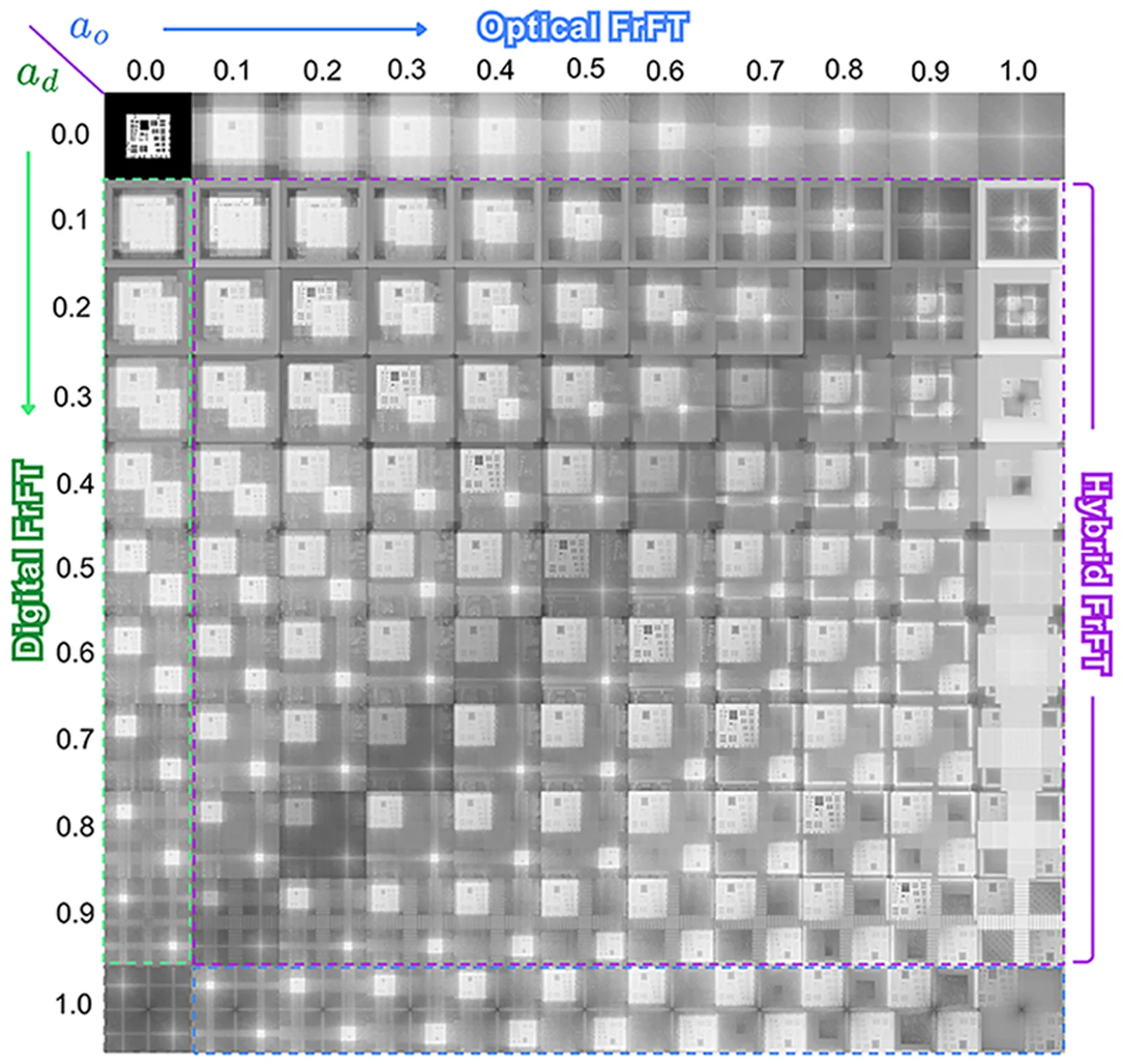
Space-frequency domain matrices of simulated off-axis holograms of the USAF-1951 target at various FrFT orders.

**Fig. 3:**
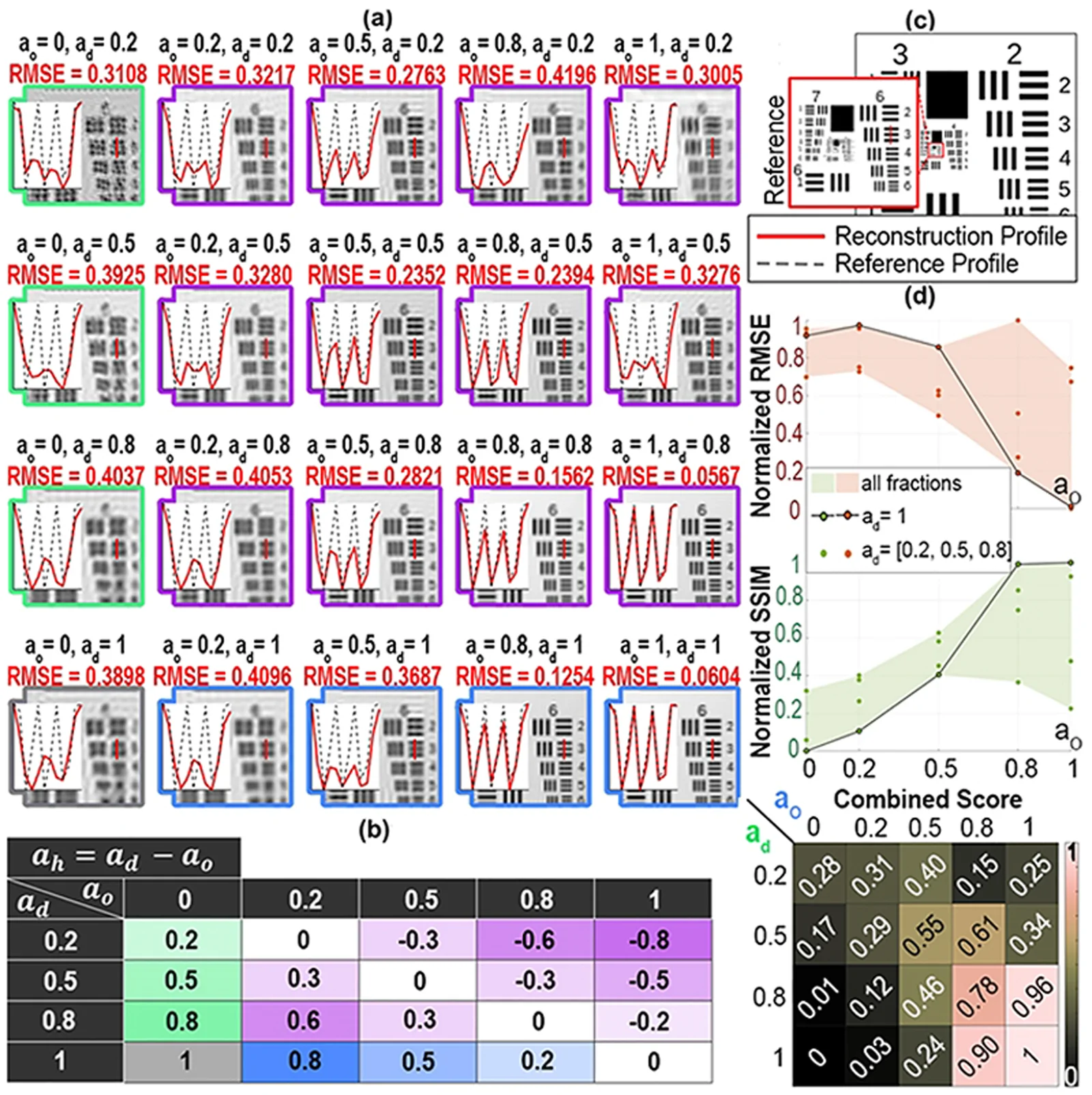
Assessment of amplitude reconstruction resolution via optical, digital, and hybrid FrFTs. (a) Profiles illustrating comparative amplitude resolutions. (b) Analysis of hybrid FrFT orders showing the effectiveness of combining optical and digital methods. (c) Illustration of the USAF 1951 target used for amplitude hologram simulations. (d) A color-coded heatmap presenting reference metrics—RMSE, SSIM, and combined score.

**Fig. 4:**
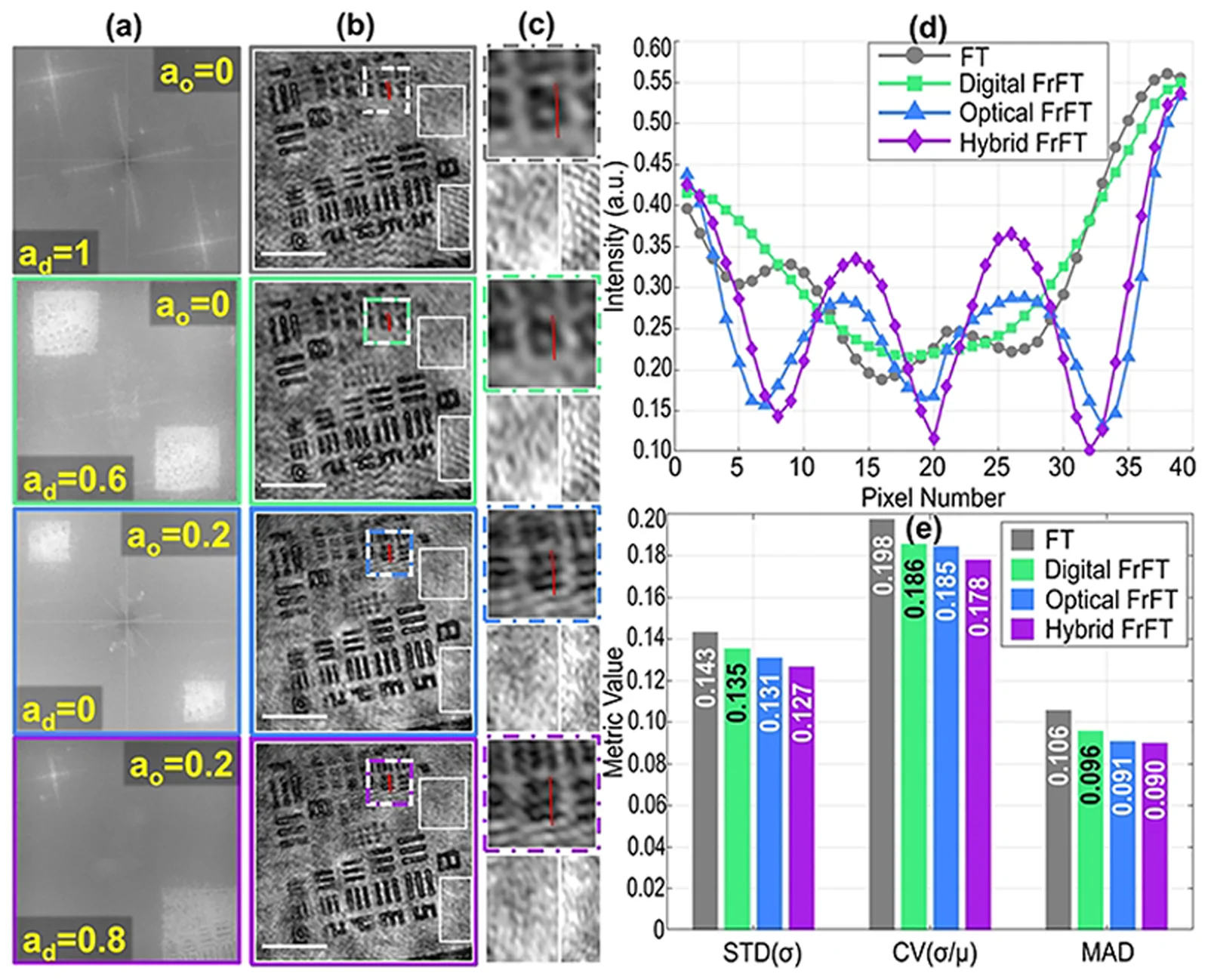
Experimental amplitude reconstruction resolution comparison of a USAF target using different FrFT combinations. (a) Space-frequency domains of recorded holograms. (b) Amplitude reconstructions. (c) Enlarged views of amplitude reconstructions used for spatial profile and deviation analysis. (d) Spatial profile plots corresponding to each FrFT combination. (e) Quantitative no-reference metrics (STD, CV, and MAD). The color codes—gray, green, blue, and purple—represent FT, digital FrFT, optical FrFT, and hybrid FrFT domains, respectively. Scale bar: 20 μm.

**Fig. 5:**
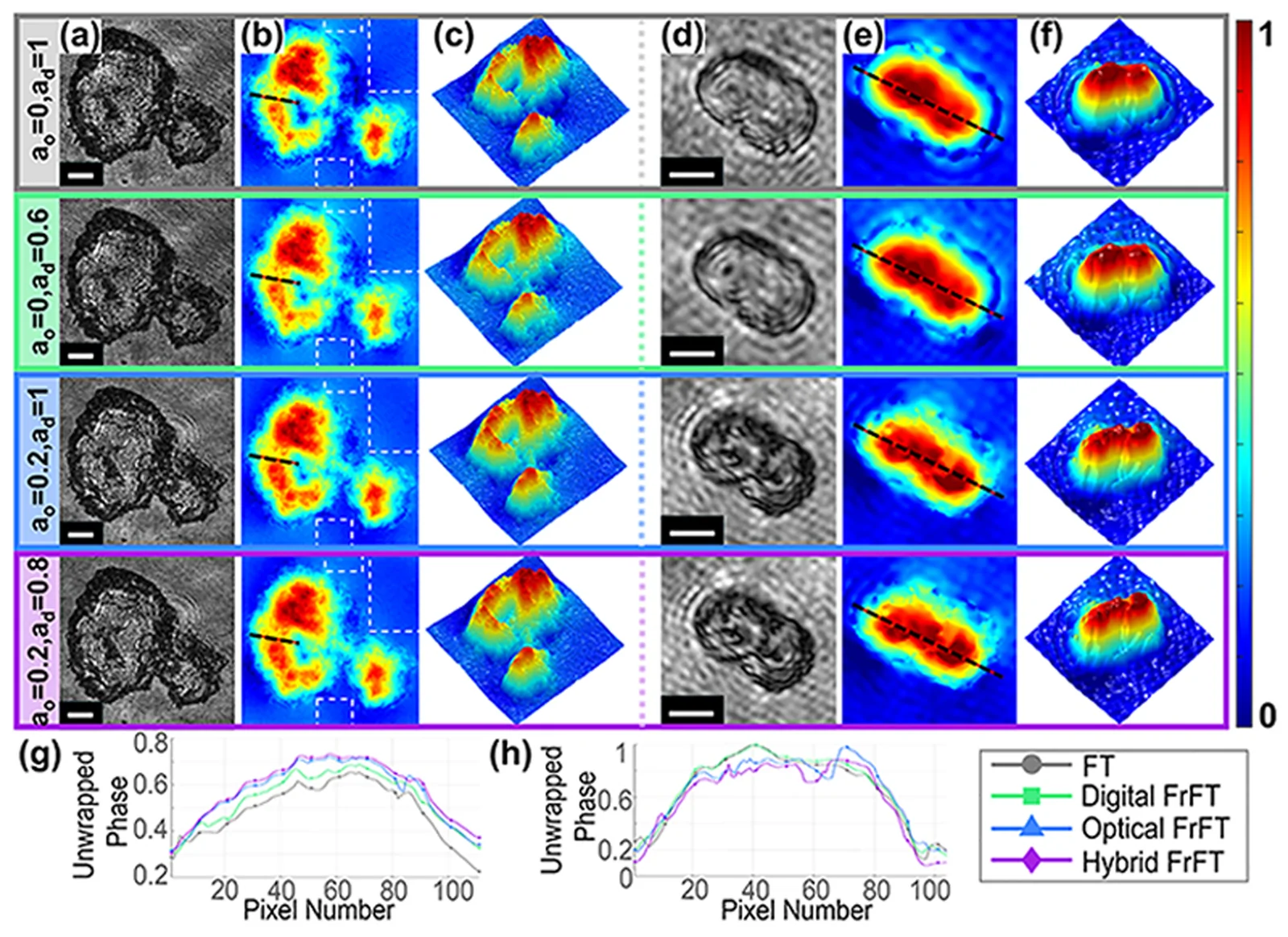
Experimental phase reconstruction resolution comparison of HT-29 cells obtained from optical, digital, and hybrid FrFT operations. (a) Amplitude, (b) phase reconstructions, and (c) pseudo-3D phase maps for four-day-old clusters. (d) Amplitude, (e) phase reconstructions, and (f) pseudo-3D phase maps for one-day-old clusters. (g) and (h) Phase profiles extracted from the dashed lines in (b) and (e). Scale bars are 20 μm (a) and 10 μm (d).

## Data Availability

The proposed method is publicly available via Bit-bucket (Code 1, Ref. [14]).
