# Supplementary material for: Hybrid fractional Fourier transform for filtering and imaging in a single digital holography workflow
Source: Opt Lett. Author manuscript; Available in PMC 2026 Oct 30. (PMC7619647; doi:10.1364/OL.575799)
Supplement: Supplementary file 1 [file EMS216049-supplement-Supplemental_document.pdf]

Supplemental Document

Optics Letters

Hybrid fractional Fourier transform for filtering and imaging in a single digital holography workflow: supplement

Muge Topcu[1,2] and Serhat Tozburun[1,2,3,*]

[1] *Izmir Biomedicine and Genome Center, Izmir 35340, Turkey*
[2] *Izmir International Biomedicine and Genome Institute, Dokuz Eylul University, Izmir 35340, Turkey*
[3] *Department of Biophysics, Faculty of Medicine, Dokuz Eylul University, Izmir, 35340, Turkey*
[*] *tozburun.serhat@gmail.com*

This supplement published with Optica Publishing Group on 2 December 2025 by The Authors under the terms of the Creative Commons Attribution 4.0 License in the format provided by the authors and unedited. Further distribution of this work must maintain attribution to the author(s) and the published article's title, journal citation, and DOI.

Supplement DOI: https://doi.org/10.6084/m9.figshare.30454625

Parent Article DOI: https://doi.org/10.1364/OL.575799

Hybrid fractional Fourier transform for filtering and imaging in a single digital holography workflow: supplemental document

MUGE TOPCU[1,2] AND SERHAT TOZBURUN[1,2,3,*]

[1]*Izmir Biomedicine and Genome Center, Izmir 35340, Türkiye*
[2]*Izmir International Biomedicine and Genome Institute, Dokuz Eylul University, Izmir 35340, Türkiye*
[3]*Department of Biophysics, Faculty of Medicine, Dokuz Eylul University, Izmir, 35340, Türkiye*
**Corresponding author: serhat.tozburun@deu.edu.tr*

1. Notation and preliminaries

Table S1. Symbols and definitions used in the supplement.

**Symbol**	**Definition**
λ	Laser wavelength
f	Focal length of the fractional lens (FL)
α; $\emptyset := \frac{\pi}{2}\alpha$	Fractional order α and rotation angle φ in the space–frequency plane
(x,y) and $\Delta x,\Delta y$	Sensor/sample plane coordinates and sampling pitches
(u, v)	Spatial-frequency coordinates (number of cycles per length)
$\mathcal{F}^{(\alpha)}\{\cdot\}$	2-D fractional Fourier transform (FrFT) of order α
$O(x,y) = \lvert O \rvert e^{j\phi_O}$	Object field at the sensor
$R(x,y) = A_R\, e^{j2\pi(f_x x + f_y y)}$	Tilted plane-wave; carrier $f_c = \sqrt{f_x^2 + f_y^2} = \sin\theta/\lambda$
DC and ± 1	Zero-order and cross terms
$N_x \times N_y$ and N_p	Sensor pixels and zero-padded grid size per dimension
NA, M, p	Numerical aperture, magnification, detector pixel pitch
a_o,a_d,a_h	Optical, digital, and hybrid fractional orders with $a_h = a_d \pm a_o$

2. Derivation of the optical recording order

The object arm consists of a microscope objective (MO), a tube lens (TL), and a fractional lens (FL). The MO and TL are positioned so that the distance between their principal planes equals $f_{\mathrm{MO}} + f_{\mathrm{TL}}$, aligning the MO's back focal plane with the TL's front focal plane. In this configuration, the TL collimates the intermediate image produced by the MO, resulting in a plane wave (apart from minor tilt or magnification effects). With this plane-wave conditioning, the FL alone implements the optical FrFT, where the fractional order depends solely on the FL's focal length, f, and the propagation distances on either side of it.

Consider a single thin lens (i.e., FL) with of focal length f. Let the input plane (the output from the MO–TL system) be located at a distance, d_1, before the FL, and the sensor (recording plane) be at a distance d_2 after the FL. As depicted in reference [1], under the standard paraxial (ABCD) model, this one-lens configuration realizes a fractional Fourier transform whose rotation angle, $\emptyset_o$, in the space–frequency plane and order a_o satisfy the following relationships.

$$\emptyset_o = \arccos\left(\frac{\sqrt{f-d_1}\sqrt{f-d_2}}{f}\right),\ a_o = \emptyset_o \frac{2}{\pi} \tag{S1}$$

Limiting cases.

- $d_1 = d_2 = f \Rightarrow \sqrt{(f-d_1)(f-d_2)}/f = 0 \Rightarrow \emptyset_o = \frac{\pi}{2}$, hence $a_o = 1$ (Fourier plane).
- $d_1 = 0$ and $d_2 = 0 \Rightarrow \varphi_o = 0$, hence $a_o = 0$ (spatial/image plane). Intermediate values of d_1,d_2 yield $0 < a_o < 1$, representing fractional planes between the image and Fourier planes.

Practical note.

The MO–TL pair sets the magnification and carrier tilt. However, by delivering a plane-wave input to the FL, it ensures that the fractional order of the optical recording is determined solely by the parameters (f,d_1,d_2) as outlined in Eq. (S1). This approach isolates order control to a single element (FL), simplifying both design and calibration processes (Fig. S1).

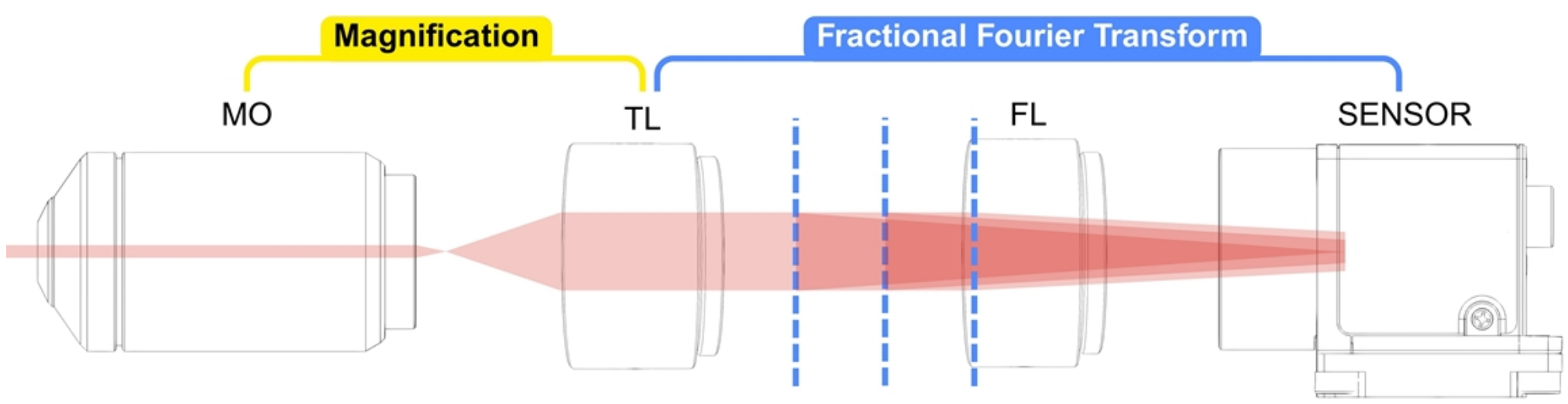

Fig. S1. MO-TL-FL three-lens object arm design that enables separation of magnification and fractional Fourier transform operations.

3. Formation of holograms in the fractional plane

Let

$$\boldsymbol{O}(\boldsymbol{x},\boldsymbol{y}) = |\boldsymbol{O}(\boldsymbol{x},\boldsymbol{y})| \boldsymbol{e}^{\boldsymbol{j}\boldsymbol{\phi}_{\boldsymbol{O}}(\boldsymbol{x},\boldsymbol{y})}, \boldsymbol{R}(\boldsymbol{x},\boldsymbol{y}) = \boldsymbol{A}_{\boldsymbol{R}} \boldsymbol{e}^{\boldsymbol{j2\pi}(\boldsymbol{f}_{\boldsymbol{x}}\boldsymbol{x}+\boldsymbol{f}_{\boldsymbol{y}}\boldsymbol{y})} \tag{S2}$$

The optical FrFT of order a_o is defined as

$$O_{a_o}(x,y) := \mathcal{F}^{(a_o)}\{O\}(x,y) \tag{S3}$$

The recorded (sampled) intensity can then be expressed as

$$\begin{aligned} I_{a_o}(x,y) &= |R(x,y) + O_{a_o}(x,y)|^2 \\ &= |R|^2 + |O_{a_o}|^2 + R^*(x,y)\, O_{a_o}(x,y) + R(x,y)\, O^*_{a_o}(x,y) \end{aligned} \tag{S4}$$

The last two terms correspond to the ±1 diffraction orders.

Translation variance in the FrFT plane. In the FrFT domain, a lateral shift of the object does not correspond to a simple shift of O_{a_o}; instead, it introduces a chirped shift whose characteristics depend on the fractional order a_o. The carrier displacement from the DC component can be expressed as

$$\mathrm{D}(a_d) = (D_x,D_y) = \left(f_x \sin(\pi a_d/2), f_y \sin(\pi a_d/2)\right) \tag{S5}$$

The diffraction-order locations in the a_d-plane are therefore shifted approximately by $\pm \mathrm{D}(a_d)$ and acquire quadratic phase factors [2]. This behavior necessitates recalibration of the region of interest (ROI) coordinates whenever a_d (or a_o) changes, and a finite sensor window in the FrFT plane back-propagates as a sinc-like apodization after inverse operations.

4. Digital FrFT, hybrid orders, and asymmetric propagation

Applying a digital FrFT of order a_d to (S4) yields

$$\mathcal{F}^{(a_d)}\{I_{a_o}\} = \mathcal{F}^{(a_d)}\{|R|^2 + |O_{a_o}|^2\} + \mathcal{F}^{(a_d)}\{R^* O_{a_o}\} + \mathcal{F}^{(a_d)}\{R\, O_{a_o}^*\} \tag{S6}$$

Using the composition and conjugation properties of the FrFT,

$$\mathcal{F}^{(a_d)}\{O_{a_o}\} = \mathcal{F}^{(a_d+a_o)}\{O\},\ \mathcal{F}^{(a_d)}\{O_{a_o}^*\} = (\mathcal{F}^{(a_d-a_o)}\{O\})^* \tag{S7}$$

Hence, the effective (hybrid) fractional orders governing the signal and its conjugate are

$$a_h^{(+1)} = a_d + a_o, a_h^{(-1)} = a_d - a_o \tag{S8}$$

The two conjugate terms thus propagate in opposite fractional directions: the $+1$ term advances by $+a_o$, whereas the -1 term reverses by $-a_o$ (Fig. S2).

Since the reference wave R is a tilted plane wave, its FrFT is analytically known to be a chirped impulse. Therefore, R or R^* acts purely as a carrier that sets a predictable phase tilt and spatial displacement without affecting the object's hybrid fractional order.

5. Filtering, back-rotation, and complex field recovery

Let $H_{a_h}(m,n)$ denote a rectangular ROI in the a_h-plane, centered at the chosen ± 1 diffraction order, with dimensions (h_x,h_y) on an $N_p \times N_p$ grid:

$$H_{a_h}(m,n) = \begin{cases} 1, & |m - m_0| \le \frac{h_x}{2}, \quad |n - n_0| \le \frac{h_y}{2}, \\ 0, & otherwise. \end{cases} \tag{S9}$$

The filtered spectrum is obtained as $I_{a_h}^{(\mathrm{filt})} = I_{a_h} \cdot H_{a_h}$.

To back-rotate the spectrum to the true spatial-frequency domain (undoing the hybrid rotation for the $+1$-order):

$$\tilde{S}(u,v) = \mathcal{F}^{(1-a_h^{(+1)})}\{I_{a_h}^{(\mathrm{filt})}\} \tag{S10}$$

After re-centering the carrier to $(u,v) = (0,0)$, inverse FT yields the complex field:

$$\Psi(x,y) = \mathcal{F}^{-1}\{\tilde{S}(u,v)\} \tag{S11}$$

For optional axial propagation using the angular spectrum method:

$$H_z(k_x,k_y) = e^{jz\sqrt{k^2-k_x^2-k_y^2}}, k = \frac{2\pi}{\lambda}, \Psi_z = \mathcal{F}^{-1}\{\mathcal{F}\{\Psi\}\, H_z\} \tag{S12}$$

Finally, the amplitude and phase distributions are obtained as

$$(x,y) = |\Psi_z(x,y)|, \phi(x,y) = \arg\{\Psi_z(x,y)\}\ (\text{unwrap } \phi) \tag{S13}$$

6. Sampling, aliasing, and the fractional lens design criteria

To prevent high-frequency undersampling, the focal length, f, of the fractional lens is selected such that the projected fractional bandwidth remains within the detector's Nyquist region. Given a pixel pitch Δx and an array size N, the spatial and Fourier-domain extents satisfy the standard DFT grid scaling [1].

$$S_{\max} = \frac{1}{4\Delta x}, F_{\max} = \frac{N\Delta x}{2\lambda f} \tag{S14}$$

Requiring $F_{\max} > S_{\max}$ leads to the design inequality:

$$f < \frac{2N\,(\Delta x)^2}{\lambda} \tag{S15}$$

This condition ensures sufficient sampling of high spatial frequencies. For example, in our experiments with $\lambda = 635$ nm, $\Delta x = 5.3\ \mu$m, $N = 1024$, the criterion gives $f < 90$ mm; thus, a fractional lens with $f = 75$ mm was appropriately chosen.

7. Effect of hybrid FrFT on improving spectral separation

Equation (S9) shows that $+1$ and -1 propagate under different hybrid orders. By jointly tuning a_o and a_d, the space–frequency content can be rotated so that the +1-order is positioned farther from both the DC and the -1-order, while remaining within the Nyquist limit. The center shift magnitude for a given digital order is

$$\| \mathrm{D}(a_d) \| = f_c \sin\left(\frac{\pi a_d}{2}\right), \qquad 0 \le a_d \le 1 \tag{S16}$$

with $\| \mathrm{D}(0) \| = 0$ in the spatial plane and $\| \mathrm{D}(1) \| = f_c$ in the Fourier plane. Maximizing the separation without exceeding the Nyquist limit enlarges the usable ROI, preserves more high-frequency content, and improves resolution, as illustrated in Fig. S2.

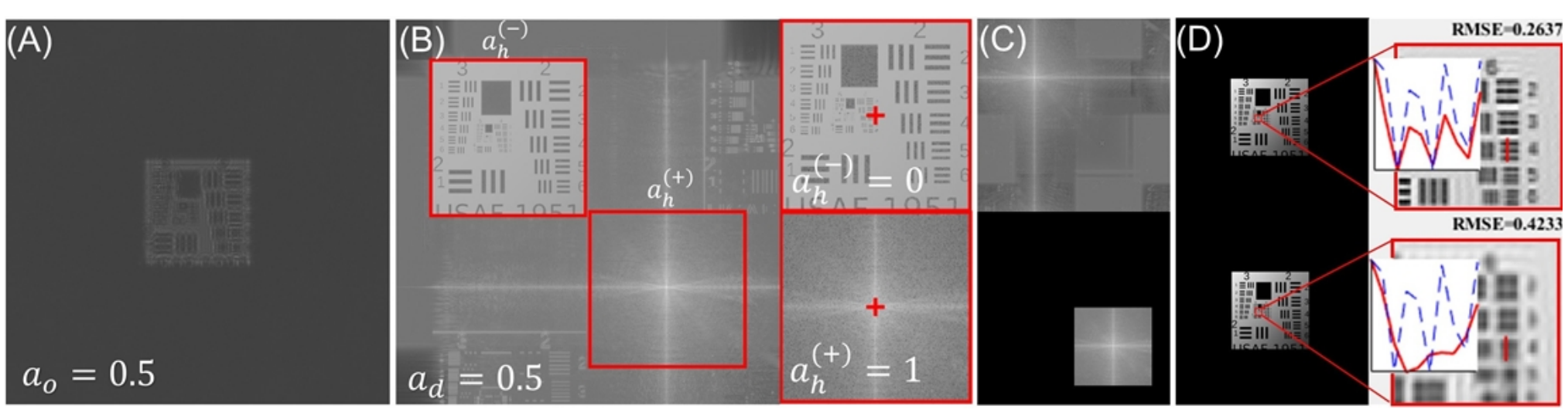

Fig. S2. Effect of asymmetric conjugation on hologram reconstruction. (A) Fractional Fourier domain hologram of a simulated USAF target with $a_o = 0.5$. (B) Fractional Fourier domain filtered hologram at $a_d = 0.5$. Resulting -1 diffraction order $a_h^{(-1)} = a_d - a_o = 0$ (image plane) and +1 diffraction order $a_h^{(+1)} = a_d + a_o = 1$ (fourier plane). (C) Filtered diffraction orders shifted to Fourier domain by $\mathcal{F}^{(1-a_h)}$. (D) Amplitude reconstructions of filtered diffraction orders by $\mathcal{F}^{(-1)}$ and their plot profiles in the zoomed region at line pair element Group 6-line pair four evaluated with root mean square error (RMSE).

8. Laplacian convolution

The discrete 3×3 Laplacian

$$L = \begin{bmatrix} 0 & -1 & 0 \\ -1 & 4 & -1 \\ 0 & -1 & 0 \end{bmatrix} \Leftrightarrow W_L(f_x,f_y) = -4[\sin^2(\pi\Delta x f_x) + \sin^2(\pi\Delta y f_y)] \tag{S23}$$

acts as a high-pass filter. When applied prior to the digital FrFT,

$$\mathcal{F}^{(a_d)}\{L * I_{a_o}\} = W_L(f_x,f_y)\,\mathcal{F}^{(a_d)}\{I_{a_o}\} \tag{S24}$$

it attenuates low-frequency DC growth that would otherwise spread under fractional rotation and encroach on the ±1 diffraction orders for intermediate a_d. In practice, this:

- suppresses the DC component while preserving energy near $\pm\,\mathrm{D}(a_d)$,
- improves order separation,
- allows larger ROIs (h_x,h_y), increasing the retained signal bandwidth.

Thus, Laplacian filtering enables the hybrid FrFT to fully realize its separation advantage (Eqs. S8 and S16). However, it is not responsible for the resolution enhancement itself, as noted in [3]. Fig. S3 illustrates amplitude reconstructions with and without Laplacian convolution.

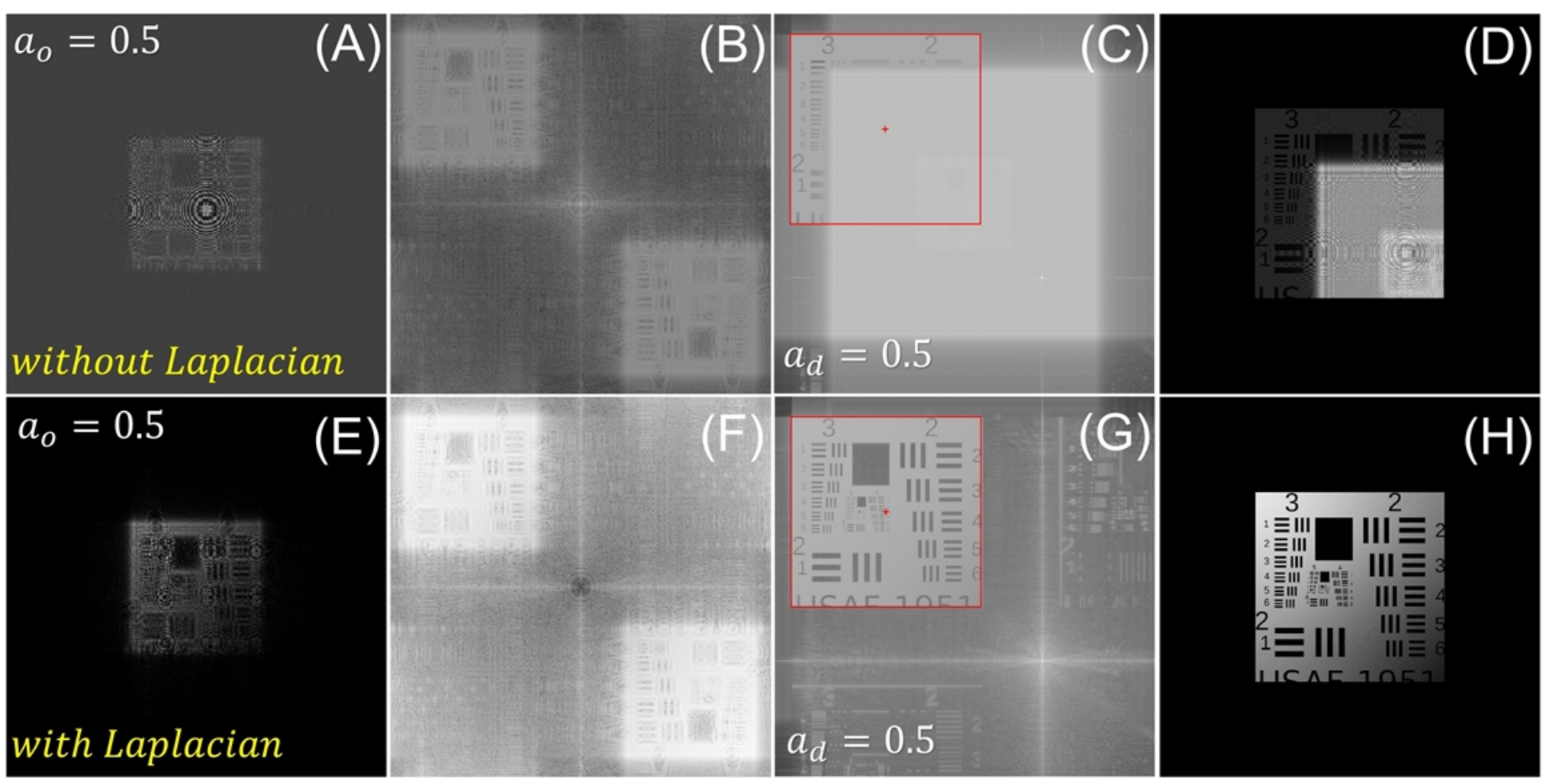

Fig. S3. Effect of Laplacian convolution on Hybrid FrFT reconstruction shown in simulated holograms with orders $a_o = 0.5, a_d = 0.5$. (A) and (E) are the simulated holograms with and without Laplacian convolution, (B) and (F) are the Fourier domains of the holograms ($a_d = 1$), (C) and (G) fractional Fourier domain filtered holograms at $a_d = 0.5$ and masked -1 diffraction order $a_h^{(-1)}$ (D) and (H) are the amplitude images reconstructed from hybrid diffraction order $a_h^{(-1)}$.

9. Simulation workflow

Inputs: USAF-1951 pattern $O_0(\xi,\eta)$; system $(\lambda,\Delta x,\Delta y,N_p)$; reference (f_x,f_y); orders (a_o,a_d); ROI (m_0,n_0,h_x,h_y); zero-padding N_p.

1. **Object generation:** Begin with the object in the object plane $O_0(\xi,\eta)$. Map and scale to sensor coordinates $O(x,y)$, taking into account magnification. Optionally, apply zero-padding to an $N_p \times N_p$ matrix.

2. **Optical FrFT (recording):** Compute $O_{\alpha_o} = \mathcal{F}^{(\alpha_o)}\{O\}$.

3. **Reference:** Create the reference $R(x,y) = A_R e^{j2\pi(f_x x + f_y y)}$ where $(f_x,f_y) = (\sin\theta_x/\lambda, \sin\theta_y/\lambda)$.

4. **Hologram:** Capture the intensity $I_{\alpha_o} = |O_{\alpha_o} + R|^2$ and sample it by using Δ_x,Δ_y on a grid of size $N_x \times N_y$. Then, zero-pad to an $N_p \times N_p$.

5. **Laplacian Convolution:** $\boldsymbol{L} * \boldsymbol{I}_{\boldsymbol{a_o}}$(Eq. S24) (Optionally adopted for zero order suppression).

6. **Digital FrFT:** Compute $I_{\alpha_h} = \mathcal{F}^{(\alpha_d)}\{I_{\alpha_o}\}$.

7. **Spectral shift and ROI filter:** Locate the +1-order center (displacement $D(\alpha_d)$) and apply a window H_{α_h} (Eq. S10) of size $h_x \times h_y$, resulting in the filtered intensity $I_{\alpha_h}^{(\mathrm{filt})}$.

8. **Back-rotation & re-center:** Back-rotate the filtered order re-center the carrier to DC component to obtain $\tilde{S} = \mathcal{F}^{(1-a_h^{(+1)})}\{I_{a_h}^{(\mathrm{filt})}\}$.

9. **Inverse FT:** Estimate the complex field using $\Psi = \mathcal{F}^{-1}\{\tilde{S}\}$.

10. **Axial propagation & focus:** Propagate Ψ using the angular spectrum H_z to the focus plane, z, maximizing a chosen focus metric (such as energy concentration or variance) to produce Ψ_z.

11. **Amplitude/phase extraction:** Extract amplitude $A = |\Psi_z|$, and phase $\phi = arg\ (\Psi_z)$, followed by phase unwrapping if necessary.

Note. The implementation uses a 2-D frft function adopted from A. Bultheel [4]; see repository script frft22d.m.

References

1. J. Zhang, Y. Liu, W. Cui, and Z. Jiang, Fractional Fourier-transform off-axis digital holographic imaging, Appl. Opt. **63**, 3922 (2024).
2. Y. Liu, Q. Sun, H. Chen, and Z. Jiang, Fractional Fourier-transform filtering and reconstruction in off-axis digital holographic imaging, Opt. Express **31**, 10709 (2023).
3. W. Cui, Y. Liu, J. Zhang, Y. Han, and Z. Jiang, Single-frame reconstruction by fractional Fourier-transform domain filtering in off-axis digital holographic microscopy, Opt. Continuum **2**, 2500 (2023).
4. A. Bultheel and H.E.M. Sulbaran, Computation of the fractional Fourier transform, App. Com. Harmon. Anal. **16**, 182-202 (2004).
